# Supplementary material for: The genome sequence of the Small Scabious Mining Bee, Andrena marginata Fabricius, 1776
Source: Wellcome Open Res. 2024 Aug 12;9:447. [Version 1] doi: 10.12688/wellcomeopenres.22772.1 (PMC11499740; doi:10.12688/wellcomeopenres.22772.1)
Supplement: Peer review history

# Reviewer report: Reviewer response for version 1

**DOI:** 10.21956/wellcomeopenres.25076.r103561

**Published:** 2024-10-23

**Authors:** Pilar De la Rúa

**Affiliations:** 1 University of Murcia, Murcia, Spain

This is a detailed article on the process of obtaining the genome of a bee species, Andrena marginata, and its main characteristics. This bee is of interest as it is considered rare and declining in the UK, so it makes sense to sequence it and preserve its extracted DNA.

 I just had a question while reading this paper, was the specimen analysed directly frozen in the field where it was collected? This is important especially when it comes to preserving the RNA and ensuring sufficient quality to be sequenced.

Are sufficient details of methods and materials provided to allow replication by others?

Yes

Is the rationale for creating the dataset(s) clearly described?

Yes

Are the datasets clearly presented in a useable and accessible format?

Yes

Are the protocols appropriate and is the work technically sound?

Yes

Reviewer Expertise:

apidologist

I confirm that I have read this submission and believe that I have an appropriate level of expertise to confirm that it is of an acceptable scientific standard.

# Reviewer report: Reviewer response for version 1

**DOI:** 10.21956/wellcomeopenres.25076.r103559

**Published:** 2024-10-23

**Authors:** Rodolpho Menezes

**Affiliations:** 1 State University of Santa Cruz, Ilhéus, Brazil

The data note by Mitchell and Monks presents a genome assembly from the Small Scabious Mining Bee, *Andrena marginata* Fabricius, 1776. The genome sequence has a total length of 373.60 megabases. This genome information will be interesting for comparative genomics investigation and also for evolution and conservation studies. The methodology is thorough and explained and the figures are well constructed, and the data note is well written.

 I believe the authors should provide more detailed information about the mitogenome, as its size of 21.16kb is relatively large compared to other hymenopterans.

Are sufficient details of methods and materials provided to allow replication by others?

Yes

Is the rationale for creating the dataset(s) clearly described?

Yes

Are the datasets clearly presented in a useable and accessible format?

Yes

Are the protocols appropriate and is the work technically sound?

Yes

Reviewer Expertise:

Cytogenetics, Phylogenomics, Molecular Systematics, and Phylogeography.

I confirm that I have read this submission and believe that I have an appropriate level of expertise to confirm that it is of an acceptable scientific standard.
